# Force Spectroscopy Measurements Show That Cortical Neurons Exposed to Excitotoxic Agonists Stiffen before Showing Evidence of Bleb Damage

**DOI:** 10.1371/journal.pone.0073499

**Published:** 2013-08-30

**Authors:** Shan Zou, Roderick Chisholm, Joseph S. Tauskela, Geoff A. Mealing, Linda J. Johnston, Catherine E. Morris

**Affiliations:** 1 Measurement Science and Standards, National Research Council Canada, Ottawa, Ontario, Canada; 2 Human Health Therapeutics, National Research Council Canada, Ottawa, Ontario, Canada; 3 Neuroscience, Ottawa Hospital Research Institute, Ottawa, Ontario, Canada; LAAS-CNRS, France

## Abstract

In ischemic and traumatic brain injury, hyperactivated glutamate (N-methyl-D-aspartic acid, NMDA) and sodium (Nav) channels trigger excitotoxic neuron death. Na^+^, Ca^++^ and H_2_O influx into affected neurons elicits swelling (increased cell volume) and pathological blebbing (disassociation of the plasma membrane’s bilayer from its spectrin-actomyosin matrix). Though usually conflated in injured tissue, cell swelling and blebbing are distinct processes. Around an injury core, salvageable neurons could be mildly swollen without yet having suffered the bleb-type membrane damage that, by rendering channels leaky and pumps dysfunctional, exacerbates the excitotoxic positive feedback spiral. Recognizing when neuronal inflation signifies non-lethal osmotic swelling versus blebbing should further efforts to salvage injury-penumbra neurons. To assess whether the mechanical properties of osmotically-swollen versus excitotoxically-blebbing neurons might be cytomechanically distinguishable, we measured cortical neuron elasticity (gauged via atomic force microscopy (AFM)-based force spectroscopy) upon brief exposure to hypotonicity or to excitotoxic agonists (glutamate and Nav channel activators, NMDA and veratridine). Though unperturbed by solution exchange per se, elasticity increased abruptly with hypotonicity, with NMDA and with veratridine. Neurons then invariably softened towards or below the pre-treatment level, sometimes starting before the washout. The initial channel-mediated stiffening bespeaks an abrupt elevation of hydrostatic pressure linked to NMDA or Nav channel-mediated ion/H_2_O fluxes, together with increased [Ca^++^]_int_-mediated submembrane actomyosin contractility. The subsequent softening to below-control levels is consistent with the onset of a lethal level of bleb damage. These findings indicate that dissection/identification of molecular events during the excitotoxic transition from stiff/swollen to soft/blebbing is warranted and should be feasible.

## Introduction

In cerebral ischemia and trauma, overactivated voltage-gated Na^+^ (Nav) and N-methyl-D-aspartic acid (NMDA) glutamate channels are responsible for the excitotoxic demise of injured neurons. In experimental models, inhibitors of Nav and NMDA channels attenuate, postpone or prevent anoxic depolarization, and hypoxia/ischemia-induced demise of neurons; likewise for mechanically-traumatized neurons [1–11]. Dysregulation of excitatory channels engenders [Ca^++^]_int_ levels deemed “excitotoxic” because they overactivate Ca^++^-proteases [12] and Ca^++^-lipases [13], with calpain-cleaved spectrin products [14] being an unequivocal correlate of irreversible excitotoxic membrane damage. In erythrocytes, it is well recognized that plasma membrane integrity and functionality of integral membrane proteins depends on robust and extensive adhesive contacts between spectrin-actin membrane skeleton components and the bilayer. In excitable cells, by contrast, membrane cytomechanics have received little attention, though where membrane skeleton/bilayer adhesions have suffered damage, various excitable cell channels are known to become leaky [15–17], i.e., in blebbed membranes. And while neuronal swelling and blebbing (or beading) are widely reported in injured and ischemic nerve tissues, it remains unclear when these changes constitute simple osmotic swelling and when they signify bleb damage in its various guises. As a start in addressing this, we have tested AFM-based force spectroscopy on cultured cortical neurons. We show that this approach can detect potentially interesting early (minutes rather than hours) changes in the cytomechanical status of neurons exposed to excitotoxic stimuli.

Membrane skeletons include dynamic ATP-dependent actomyosin elements that interact with more stable spectrin-based meshworks. While ankyrins connect spectrin to various transmembrane membrane proteins (e.g., Nav channels [18]), a multitude of weaker non-specific interactions bind membrane skeleton proteins to inner leaflet bilayer lipids [19–24]. The ensemble of non-covalent interactions yields a mesoscopic level of mechanical adhesion, stabilizing the plasma membrane bilayer against rupture or vesicle shedding and against the decay of its healthy, dynamic leaflet asymmetry [25] and lateral heterogeneity. Blebbing - the chemically- and/or mechanically-induced [26] pathological diminishment of these adhesive contacts - leads to necrosis, microvesicle shedding or apoptosis [27]. Confusingly, the healthy physiological process of locomotory protrusion has also been labeled “blebbing”, but there, cortical F-actin repeatedly attaches/detaches from a retained spectrin-based skeleton [28]. By contrast, in sick-cell (pathological) blebbing, both actomyosin [17,29] and spectrin detach [30,31] and the biologically structured bilayer denatures [32] towards a self-organized equilibrium structure of minimal energy, maximal entropy.

Embedded in these denaturing bilayers are functioning, though possibly misbehaving, membrane proteins [13,17,29,33,34]. In damaged excitable cell membranes, both NMDA channels and voltage-gated channels (including Cav and Nav and K) are considered “leaky”; they activate too easily [15,35–39]. In sick excitable cells, progressive bleb-damage would therefore contribute to lethal excitotoxic cascades [40,41]. In experimental stroke, even with NMDA channels blocked, the Nav-rich axon initial segments of cortical neurons suffer profound excitotoxic membrane damage (as evidenced by Ca^++^-protease fragments of spectrin) [20]: presumably, ATP depletion fosters a vicious cycle of rising [Na^+^]_int_ and [Ca^++^]_int_, thence bleb-damage and further Nav-leak and ATP depletion [40].

Here, we addressed the feasibility of monitoring the onset of excitotoxicity from a non-invasive cytomechanical perspective. Excitotoxic agonists depolarize neurons. Do they elicit in neurons the response seen in vascular endothelial cells during high-[K^+^]_out_-induced depolarization [42], namely, a monotonic softening? Excitotoxic agonists dilate neurons. Do they elicit in neurons an increase in elasticity, consistent with a hydrostatic pressure increase, or a softening, as expected when plasma membranes become blebbed? We found that the response of cortical neurons to high concentrations of excitotoxic agonists was biphasic: an initial stiffening followed within minutes by softening. This shows promise for future cytomechanical/cell biological studies of early events associated with excitotoxicity. But even before the molecular players are identified, AFM-based mechanical measurements could be used to non-invasively monitor excitotoxicity/neuroprotection [3] in brain slice preparations [43].

## Materials and Methods

Tissue culture plates were obtained from Du Pont-Life Technologies (Burlington, ON, Canada). Fetal bovine serum (FBS) and horse serum (HS) were from Gemini Bio (Woodland, CA, U.S.A.) and Hyclone Laboratories (Logan, UT, U.S.A.), respectively. Minimal essential medium (MEM) was from Wisent Canadian Laboratories (St-Bruno, QC, Canada). The N-methyl-D-aspartate (NMDA) receptor antagonist, MK-801, was from Tocris Bioscience (Ellisville, MO, U.S.A.). NMDA, veratridine, propidium iodide and all other reagents were from Sigma (St. Louis, MO, U.S.A.).

### Cell culture

Animal use was approved by the Animal Care Committee at the National Research Council, Canada. Neuron/glia cultures of embryonic day 18 rats were prepared as described previously [44]. One plating session (culture) per week was done for several weeks; halothane- anesthetized timed-pregnant Sprague-Dawley rats (Charles River Canada, St. Constant, QC, Canada) were sacrificed by cervical dislocation. The dissected cortical region was centrifuged (250 *g*, 5 min, 4 ^o^C) then dispersed by trituration. Culture medium was MEM supplemented to yield 25 mM glucose, 10% FBS, 10% HS. Cells were plated on poly-L-lysine coated glass coverslips or 12-well plates at 1.3 × 10^6^ cells/ml of medium, and maintained in an incubator at 37 ^o^C and 5% CO_2_. After 4 days *in vitro*, to minimize glial growth, cultures were treated with 15 μg/ml of 5-fluoro-2’-deoxyuridine and 35 μg/ml uridine. At 7 days, half the medium was replaced by MEM supplemented with 25 mM glucose and 10% HS. All experiments involved cultures from several different plating sessions, and were performed on cultures grown 14-18 days *in vitro*.

### Treatment with NMDA, veratridine and low-Na^+^ BPS

A basic physiological solution (BPS) was used, comprising: 145 mM NaCl, 5 mM KCl, 2 mM CaCl_2_, 20 mM HEPES, 0.03 mM glycine, 5 mM glucose, pH 7.4. Three different experimental treatments were used. Before treatments, cultures were washed in BPS at room temperature. Veratridine (25-50 µM ± 1 µM MK-801) and NMDA (12.5-50 µM) treatments and all wash-outs were performed in BPS. Treatments with low Na^+^ solution (± 1 µM MK-801) used a BPS variant with 70 mM NaCl (217 mosM vs 310 mosM for BPS).

### Assessment of neurotoxicity

In tandem with AFM experiments, neurotoxicity was assessed in sister cultures 24 h after treatment with NMDA and veratridine (20 min) and hypotonic solution (10 min) using the cell death marker propidium iodide (PI), as described previously [45]. In brief, medium in 12-well plates was replaced for 30 min by BPS with PI at 33 µg/ml, then fluorescence intensity (Ex= 520±20 nm; Em = 645±20 nm) was measured from four locations/well on a Cytofluor 2350 fluorescence plate reader (Millipore Corp, Bedford, MA). PI-uptake in untreated control cultures was seen in <5% of cells. The % of treated cells showing PI-uptake was assessed by subtracting PI-fluorescence in untreated (control) sister cultures, then normalizing this against 100% neuronal death (from sister cultures exposed to 100 µM NMDA for 60 min). Error bars are based on n = 6-9 for each treatment.

### Atomic force microscopy and optical imaging

Force measurements were recorded by using a JPK NanoWizard II BioAFM (JPK Instruments, Berlin, Germany) integrated with an inverted optical microscope (Olympus IX81, Olympus, Tokyo, Japan). This ensemble was enclosed in an acoustic isolation box fabricated in-house, and mounted on a Micro 60 active vibration isolation table (Halcyonics/Accurion GmbH, Goettingen, Germany). All measurements were performed in an aqueous environment; neuronal cell cultures on glass coverslips were mounted in the JPK liquid cell holder and incubated in BPS at room temperature for 1 h prior to AFM measurements. Brightfield or phase contrast optical images (40x objective) were used to locate well-separated neurons for AFM/force spectroscopy and to monitor neuronal morphology and viability during AFM experiments. All bright field images shown in the text are 224 x 168 µm^2^. Changes in cell size (perimeter) were measured using ImageJ.

Force curves were collected within a 200 x 200 nm region at the center of the neuron soma using contact mode with a 20 µm diameter borosilicate spherical glass bead mounted on a 450 ± 10 µm (length) by 50 ± 7.5 µm (width) cantilever (sQube CP-BSG-C5, Germany). Experiments were performed in BPS. The spring constants of the cantilevers were calibrated as 0.14 ± 0.03 N/m at room temperature, using the thermal noise method. Force curves were collected at a rate of 1 µm/s with applied load forces of less than 2 nN for each force curve. The resulting indentation length was controlled to be less than 1 µm, which is 20-25% of the height of the neuronal soma (typically 4-5 µm). The sample rates were set at 2048 Hz or 4096 Hz.

### Data analysis

Young’s modulus of the neuronal soma was calculated using the Hertz model [46–48] assuming a spherical incompressible AFM tip. Batch force analysis routines within the JPK image processing software were used to semi-automate the analysis of the indentation curves. The sphere radius was set at 10 µm, with a Poisson ratio of 0.5. The RMS residual values of the fits were on the order of 20-30 pN. Each data point for elasticity of control or treated neurons represents an average of at least 50 force curves.

## Results

### Baseline conditions

**Figure 1:**
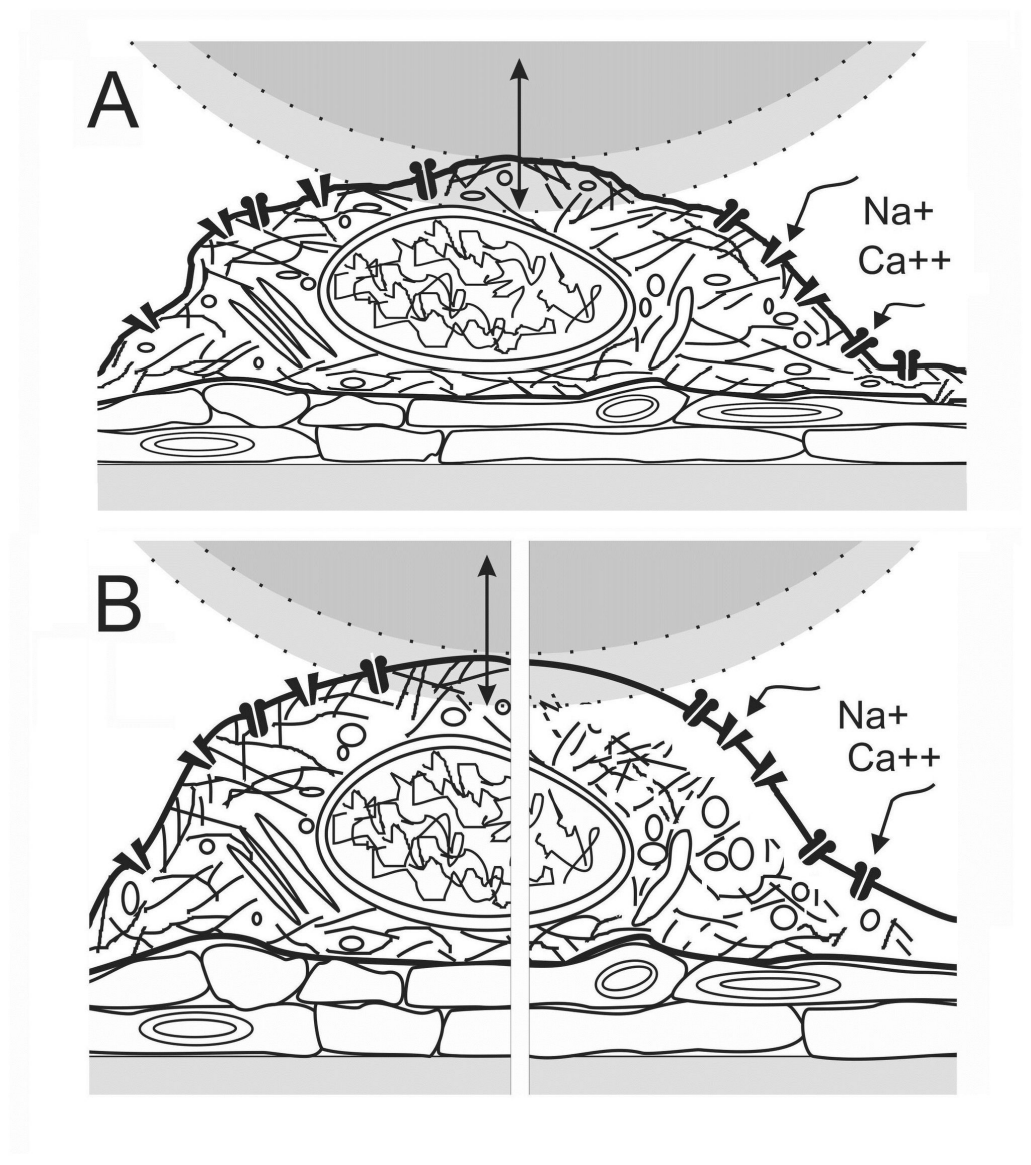
Cartoon of AFM force indentation measurements. **A**. A neuron cultured with astrocytes is depicted approximately to scale against a 20 µm sphere whose maximum downward displacement during an indent excursion is shown shaded; force is measured during “down”. Cortical neuron excitotoxicity was mimicked using agonists for glutamate and Nav channels; NMDA opens NMDA-glutamate channels, dissipating the [Ca^2+^] and [Na^+^] gradients, whereas veratridine opens Nav channels, dissipating the [Na^+^] gradient. Neurons enlarge on exposure to hypotonic medium and to excitotoxic agonists. **B**. Our expectations for the state of an enlarged neuron in the presence of excitotoxic levels of agonist is depicted at early and later stages: at left, once the initial channel-mediated Na^+^ influx and osmotically-obligated H_2_O has hydrostatically inflated the neuron (countered to some extent by Ca^2+^-mediated actomyosin contractility), and at right, after Ca^2+^-toxicity has damaged the previously adherent (and contractile) neuronal membrane skeleton, allowing the plasma membrane to bleb pathologically.

**Figure 2:**
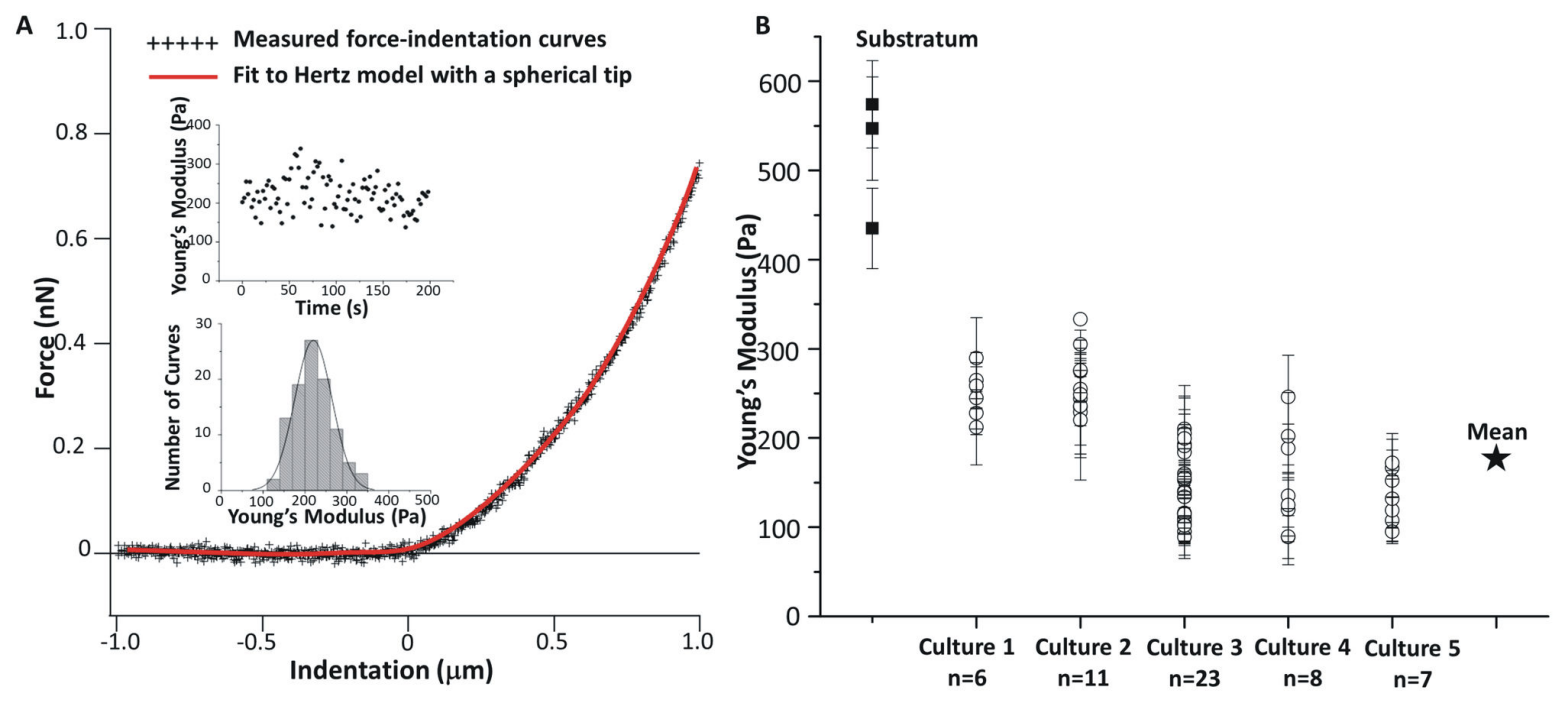
Elasticities of neuron somata. (A) Representative measured force-indentation data with a fit (red solid curve) to the Hertz model. Insets show the individual data points and histogram for 100 force-indentation data collected over a 200 s period time on the same neuron (from Cell Culture Plating 3). (B) Young’s moduli of 54 neuronal somata from five independent cell culture platings and a comparison with the background cellular substratum. The Young’s modulus varies from neuron to neuron and among different culture batches. Error bars are standard deviations for each neuron.

The mechanical properties of cortical neurons were examined by AFM-based force spectroscopy using primary co-cultures, i.e., neurons growing with astrocytes and microglia. Fluorescence and AFM images of co-cultures that were immunostained for MAP-2 and GFAP and fixed are shown in Figure S1 of the Supporting Material. The mechanical properties of the neurons were assessed under various conditions by measuring force indentation curves using a large spherical probe (Figure 1A, B). In contrast to using a small AFM probe that evaluates the local elasticity of specific regions of a cell, this method of force spectroscopy provides an average elasticity measurement for the neuronal soma. Initially the elasticity of a large number of neurons was measured in order to provide a baseline for studies of the effects of activation of NMDA and Nav channels. Individual well-separated neurons were identified by optical microscopy for measurement of force-indentation curves. A representative curve and the fit to the Hertz model [46–48] to extract Young’s modulus are shown in Figure 2A and additional approach/retraction curves are shown in Figure S2A. Analysis of 100 curves for an individual neuron (insets in Figure 2A) gave a Young’s modulus of 220 ± 45 Pa and demonstrated that the cell elasticity did not vary during data acquisition. Figure S2B illustrates the histograms obtained from force-indentation curves for 4 individual neurons from a single cell culture, illustrating the neuron-to-neuron variability. Figure 2B summarizes the Young’s modulus extracted from force-indentation curves measured for 54 neurons from 5 independent cultures over a period of several months. There was substantial variation in the Young’s modulus for individual neurons both within and between cultures, with values ranging from 80 to ~340 Pa and a mean of 177 ± 63 Pa. This was much lower than the value for the neuron’s background astrocytic matrix, whose Young’s modulus varied from 440 to 570 Pa (Figure 2B). Control experiments demonstrated the viability of the culture during the several hours required for the AFM-force spectroscopy experiments. Repeated measurements of sets of 50-100 force indentation curves on the same neuron gave similar values for Young’s modulus.

### Changes in neuronal elasticity induced by activating NMDA-glutamate channels

**Figure 3:**
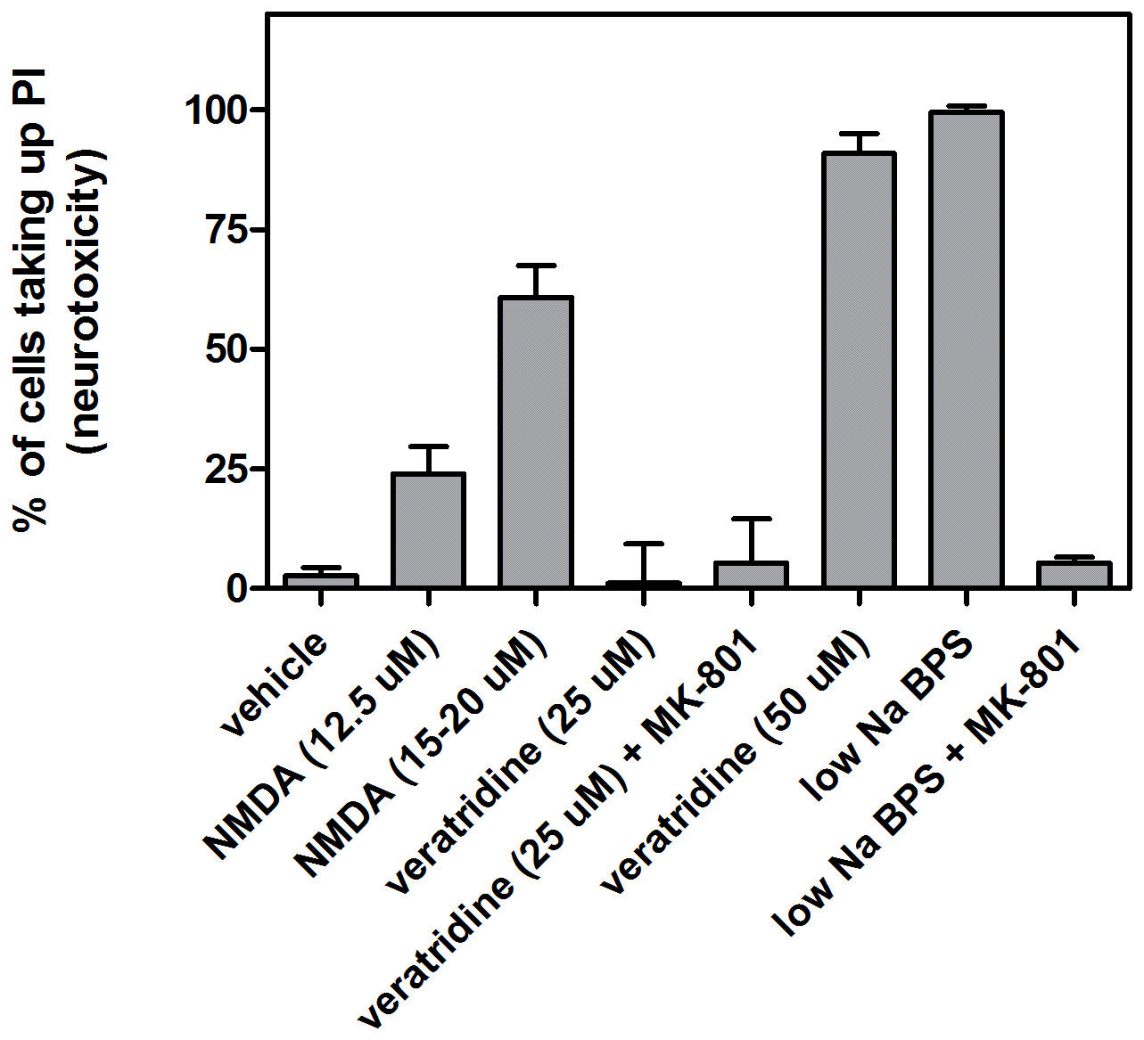
Effects of agonist treatment and hypotonic solution on neurotoxicity. Neurotoxicity was assessed using % PI-uptake 24 h after treatment. Neuronal cultures were exposed as per bar diagram labels for 20 min for NMDA and veratridine and for 10 min for low Na BPS. MK-801 prevented PI-uptake related to NMDA channel activation, both during NMDA-treatment and during low Na^+^ induced neuronal swelling. In the latter case, neurotoxicity was presumably a secondary consequence of swelling-induced glutamate release (and hence, of hyper-activation of NMDA channels). Error bars are standard deviations based on n = 6-9 for each treatment.

NMDA is an agonist that activates a nonselective cation channel (the Na^+^, K^+^ and Ca^++^ permeant NMDA-glutamate channel). Prior to investigating the mechanical effects of activation of this glutamate channel, neurotoxicity of the NMDA agonist was assessed using the cell death marker propidium iodide (PI) and agonist concentrations based on literature precedent [49]. Figure 3 demonstrates that neurons exposed to 12.5 µM NMDA showed lower neurotoxicity (25% PI-uptake after 20 min NMDA treatment) compared to 15-20 µM NMDA (63% PI-uptake). Initial investigations of the mechanical effects of NMDA treatment measured the elasticity of neurons before and after treatment, as shown in Figure S3 for 3 individual neurons. There was a clear and substantial decrease in E for each cell after NMDA treatment. Brightfield optical images measured before and after NMDA treatment demonstrated swelling of the neurons (Figure S4); the perimeter increased by an average of 30 ± 10%, based on analysis of 20 cells before and after NMDA treatment.

**Figure 4:**
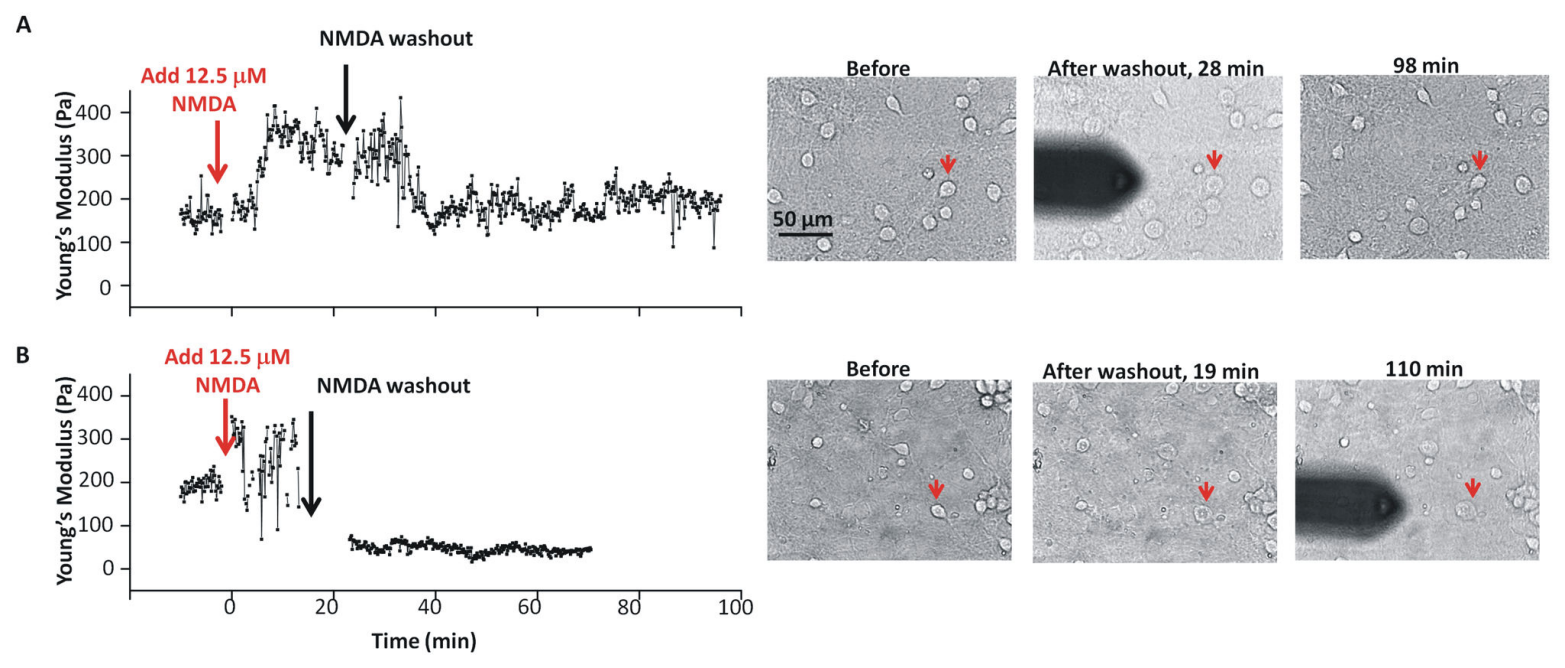
Elasticity changes as a function of time. Young’s modulus and bright field images of two neuronal somata measured as a function of time before, during exposure to 12.5 µM NMDA, and after washout. The red arrows on the optical images indicate the neurons that were measured in the indentation experiment.

To assess the mechanical effects of activation of the glutamate channel in more detail, the elasticity of cortical neurons was measured before, during and after treatment with a lower concentration (12.5 µM) of NMDA agonist. We hypothesized that early effects of channel activation might be more readily observed at concentrations of NMDA that lead to lower overall neurotoxicity. The representative time course experiments in Figure 4 for cells treated with 12.5 µM NMDA and then washed with BPS showed a biphasic response. There was a rapid increase in Young’s modulus after NMDA addition, followed by a decrease. In some cases the neuronal elasticity decreased to approximately the pretreatment level, E_0_, (Figure 4A), whereas in others it fell below E_0_ (Figure 4B). A control experiment demonstrated that BPS exchange did not lead to transient neuronal stiffening (Figure S5). Analysis of multiple time course experiments for neurons treated with 12.5 µM NMDA indicated that approximately half the neurons (4 of 7) recovered to the pretreatment value (see Figure S6 for individual experiments). With one exception the Young’s modulus increased rapidly in the presence of NMDA, with the maximum value, E_max_, attained within 2-5 min. Due to the neuron-to-neuron variability in Young’s modulus, the changes in elasticity are more readily compared by normalizing to the pretreatment value, E_0_. The maximum elasticity for individual neurons was about two to four times the pretreatment value (average E_max_/E_0_ of 2.8 ± 1.0). The time required for softening varied from neuron to neuron, but the decrease in Young’s modulus was usually complete before 25 min, independent of when washout began (this varied from 5–25 min (Figure S6)). This variability may be caused by different responses of individual neurons to NMDA, with some neurons being more sensitive and responding more rapidly than others. This result parallels the observation that only a fraction of cells shows neurotoxicity as measured by PI-uptake.

Optical images recorded at several time points during the force spectroscopy experiments indicated that addition of 12.5 µM NMDA caused most neurons within the field of view to enlarge. Optical images for the neurons used for indentation curves were obtained before NMDA addition, immediately after the NMDA washout and at the end of the experiment. Images were recorded for adjacent neurons not obscured by the AFM cantilever at various time points throughout the experiment. Neuron somata increased in size within minutes of NMDA addition and maintained their changed appearance until after washout. By the end of the experiment some neurons had recovered their original size and morphology, whereas others were still enlarged or had ruptured (Figure 4). There was a tendency for recovery of size when Young’s modulus returned to the pretreatment value after NMDA washout, and no recovery where neurons softened below the pretreatment value.

Time course experiments with 15 and 20 µM NMDA also showed early transient stiffening of neurons followed by softening. Among 8 neurons, the average elasticity increase, E_max_/E_0_, was 1.8 ± 0.3. The lower value (compared to 2.8 for 12.5 µM NMDA) may reflect more rapid softening at the higher NMDA concentrations, limiting our ability to resolve the earliest stiffening. Optical images showed that the neurons enlarged after NMDA addition. Some recovered their original size, but this had no correlation with elasticity.

**Figure 5:**
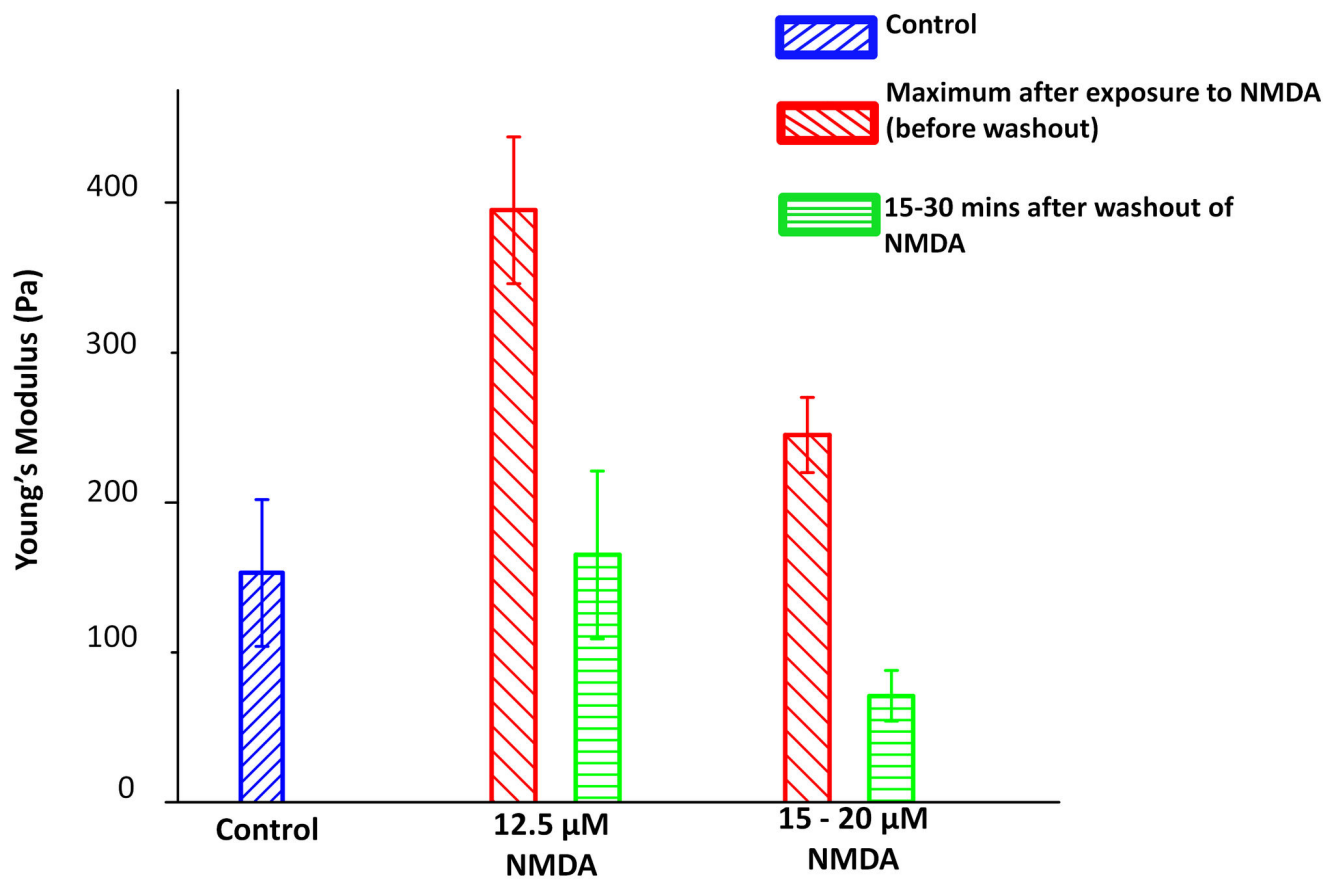
Mechanical responses of neuronal somata to exposure to NMDA. Young’s moduli of the control neurons before treatment (blue, n=11); maximum values of Young’s modulus after exposure to 12.5 µM (red, n=7) and 15-20 µM NMDA (red, n=7) before washout; and averaged values measured during the 15-30 mins after wash-out of NMDA (green). Error bars were calculated as standard errors.

The early (maximum) and later (averaged) responses to the NMDA treatments are summarized in Figure 5. The data show transient stiffening after addition of NMDA, with a larger effect for the lower concentration of NMDA. As noted above the apparently lower effect at higher NMDA concentrations may be due to a more rapid neuronal response to agonist, such that the time resolution of the force spectroscopy measurements is not sufficient to detect the initial rapid increase in elasticity. The initial stiffening is followed by a decrease in elasticity; at 12.5 µM NMDA the average Young’s modulus is similar to the initial pretreatment value while for higher concentrations the average E at later times is below E_0_ (student t-test gives a p value of 0.008 for control and 15-20 µM after washout data, indicating that the two E values are significantly different).

### Nav channel activation induces changes in neuronal elasticity

**Figure 6:**
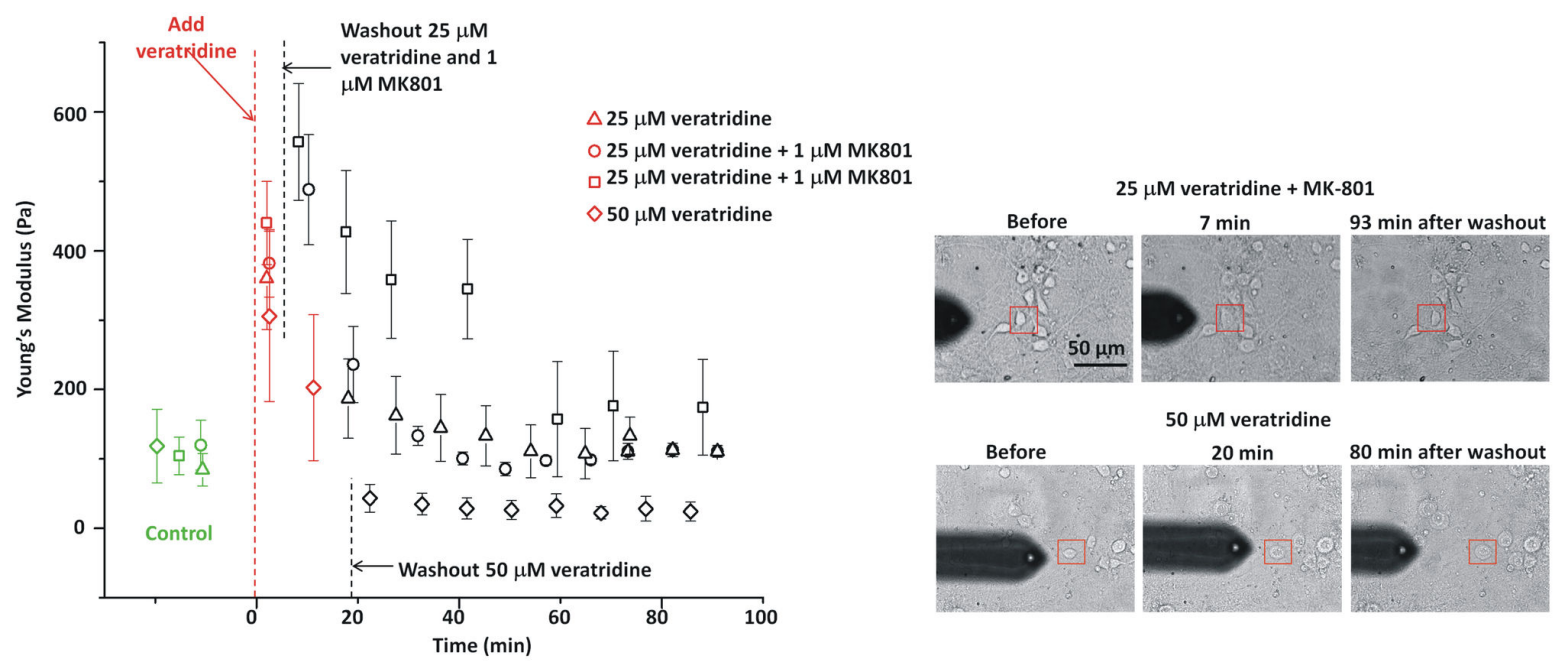
Neuronal stiffening upon exposure to veratridine. Neuronal somata “recovered” both optically and mechanically after treatment with 25 µM veratridine (with or without 1 µM MK801). The Young’s modulus of a neuron exposed to 50 µM veratridine decreased to below the initial value. Error bars are standard deviations for each neuron soma.

Nav channel leak, along with NMDA channel activation, is strongly implicated in neuronal excitotoxicity. While Nav channels themselves pass essentially no Ca^++^ [50], Nav channel activity would activate any voltage-dependent Ca^++^ channels [51]. With this in mind we also investigated the effect of the Nav channel agonist, veratridine, on neurotoxicity and the mechanical properties of neurons, selecting initial concentrations based on previous studies [52]. Figure 6 shows time course experiments for veratridine. Both 25 and 50 µM elicited a rapid increase in Young’s modulus, followed by softening of the neuron soma. For the lower concentration, Young’s modulus returned to close to its pretreatment value whereas for 50 µM veratridine it decreased below E_0_. Plasma membrane depolarization can lead to release of glutamate from neurons, which can then activate NMDA receptors [52]. Including MK801 to block NMDA channels, however, did not change the response to 25 µM veratridine. Optical images showed that veratridine-induced stiffening was accompanied by an increase in neuronal size; neuron somata subsequently recovered their original size after 25 µM veratridine, but not after 50 µM veratridine (Figure 5). In sister cultures, treatment with 50 µM veratridine was neurotoxic as measured 24 h later (Figure 3), whereas 25 µM veratridine had little effect in either the presence or absence of MK-801.

### Effects of hypotonic conditions on neuronal elasticity

**Figure 7:**
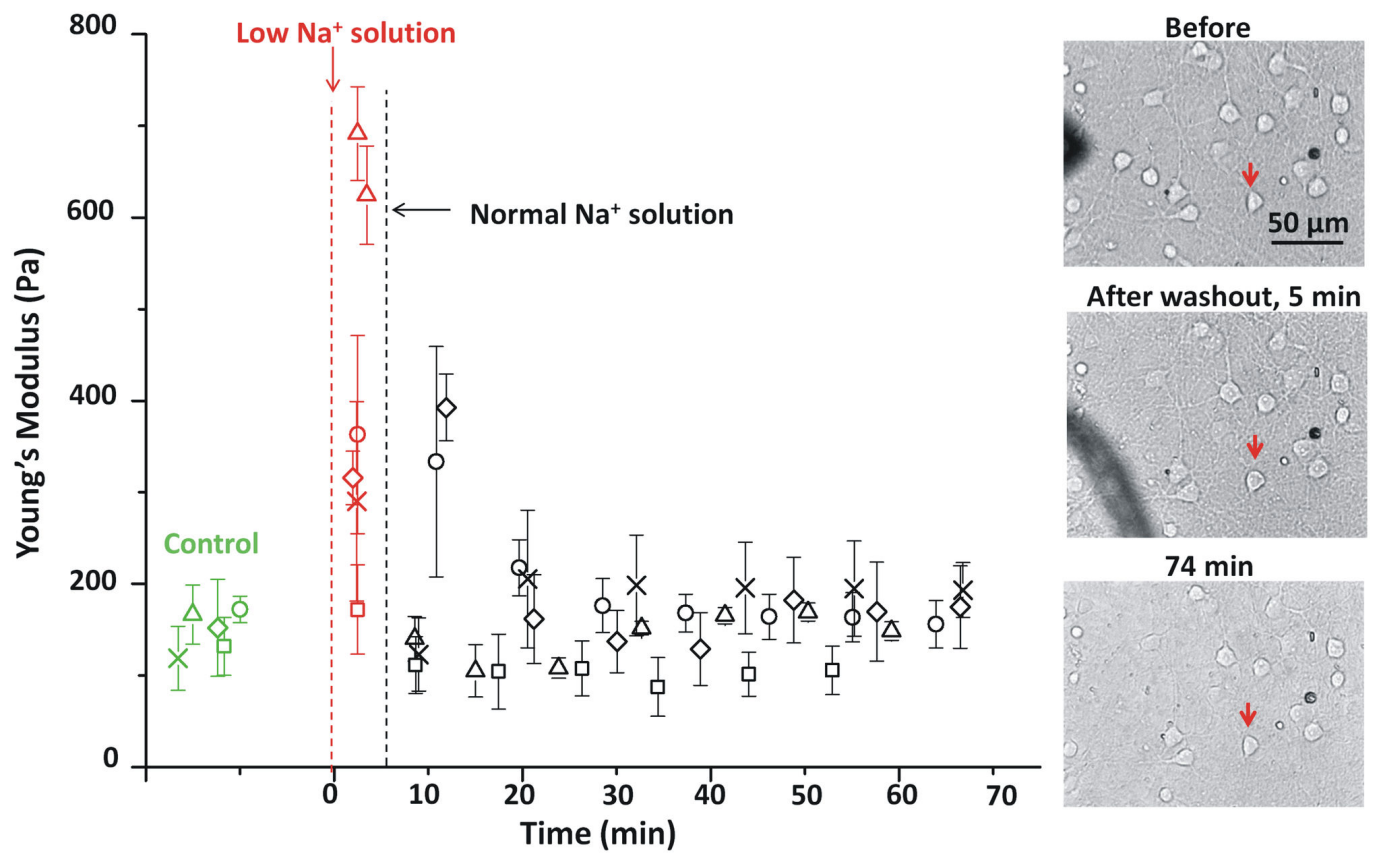
Elasticity changes in response to a brief hypotonic shock. The Young’s modulus for five neurons (each with a different symbol) are plotted before (green), during (red) and after (black) treatment with low Na^+^ BPS (70 mM, 217 mosM). The low Na^+^ BPS was replaced with BPS solution at 5 min (cross, diamond, square and circle symbols), and 7 min (triangles). Images show the morphology of the soma for one experiment (diamonds, neuron studied is marked with a red arrow) before and at two times after hypotonic shock. Error bars are standard deviations for each neuron soma.

In order to examine the cytomechanical consequences of osmotic swelling, changes in the neurons due to a 5 min exposure to a hypotonic solution were investigated. Initial PI-uptake experiments showed that treatment with hypotonic solution (BPS with reduced NaCl, 70 mM, 217 mosM) was neurotoxic whereas hypotonic BPS + MK-801 did not lead to neurotoxicity (Figure 3). The addition of the NMDA antagonist MK-801 to block secondary effects due to NMDA channel-activation [53,54] implies that hypotonic swelling leads to neurotoxic glutamate release. Replacement of BPS with hypotonic solution containing MK-801 resulted in transient stiffening of the neuronal soma (Figure 7). Neuronal elasticity decreased to the initial value on return to the original BPS solution, a process that was complete in ~20 min. Optical images (Figure 7) showed that the neurons swelled appreciably within 1-2 min after addition of hypotonic solution then recovered their original size by ~20 min after treatment (29% increase in perimeter within the first 2 min).

## Discussion

In neurons, NMDA-activated glutamate channels mediate small Ca^++^ influxes and large Na^+^ influxes and veratridine-activated Nav channels mediate Na^+^ influxes. NMDA-induced Ca^++^ influxes rapidly increase [Ca^++^]_int_ below the membrane by orders of magnitude. Agonist-induced Na^+^ influxes simultaneously depolarize neurons and draw in substantial amounts of osmotically-obliged water. During agonist and hypotonic treatments, secondary responses of voltage-gated Ca^++^ channels, other Ca^++^-permeant channels and transporters, plus intracellular Ca^++^ stores might also occur, thereby raising [Ca^++^]_int_.

As expected, exposing cortical neurons to both of the excitotoxic agonists (NMDA [55] and veratridine [56]) and to the hypotonic medium caused them to increase in size. Additionally, during the ~2 minute period between changing solutions and obtaining the next measurement, all these treatments caused somata to become stiffer. In the case of the hypotonic medium, the increased elasticity disappeared upon return to normal medium; since the neurons returned to the pre-treatment level, the simplest interpretation is that the changes in Young’s modulus reflected a reversible increase in hydrostatic pressure. Since the solution was made hypotonic by decreasing its NaCl content, it is unlikely that stiffening was associated with an increased Na^+^-influx. Agonist-induced stiffening also reversed upon washout, though time courses were more variable. For both NMDA and veratridine, it is assumed that the initial stiffening was driven by increased Na-influx plus osmotically-obliged water. Notably, softening sometimes commenced with agonist still present. Force indentation experiments for neurons treated with 25-50 µM NMDA showed an initial stiffening followed by irreversible softening indicative of an irreversible alteration of their cell membrane mechanics. The secondary stage – irreversible softening - we take to signify that the neuron had suffered severe bleb-type membrane damage, as explained in the next section. As judged 24 h later from sister cultures to those prepared (at the same time) for AFM-experiments, 12.5 µM NMDA and 50 µM veratridine were lethally neurotoxic for 30 min exposures, causing ~25% and ~95% kill, respectively. At 12.5 µM NMDA the indentation data showed on average, return to pre-treatment elasticity but that average includes some neurons that softened to below-control levels (as in Figure 4B) consistent with the onset of a lethal level of bleb damage.

In neurons, including the cortical neurons studied here, bleb damage does not yield clear fluid-filled ballooned structures like those typically seen in undifferentiated cell lines (e.g. [57]) or in some fully-differentiated cells (e.g., inner hair cells [58], skeletal muscle fibers [33]). Once formed, these balloon blebs are typically not retrieved. In response to hyposmotic stress, anoxia, stretch, excitotoxic agonists and other noxious conditions, neuronal somata bulge and distend without producing ballooned bilayer evaginations [59,60]. For elongated neural processes (neurites, axons, dendrites) the blebbing response takes the form of beading (also called pearling) [3]. Beading results from the deterioration of bilayer/membrane skeleton adhesions [61] and can, in some cases, be reversible [62,63]. Importantly, however, gross morphological reversibility might not return the neurolemma and its proteins to their pre-insult state; native stretch-activated K channels in molluscan neurons, for example, remain hyper-sensitive to membrane stretch many hours after a hyposmotic insult [64].

The findings presented here now show that, for cortical neuron somata, severe bleb-type damage arising from exposure to excitotoxic agonists is detectable immediately as a cytomechanical change, in which the soma first stiffens. The average cytomechanical response to NMDA was biphasic, with stiffening followed within minutes by a subsequent softening to below pre-treatment elasticity for 25-50 µM NMDA. The biphasic pattern in response to excitotoxic agonists suggests that initially, the as-yet uncompromised plasma membrane sustained osmotic stress due to the influxes of Na^+^ and water. Anion influx through volume sensitive chloride channels may also have increased [65]. As the neurons depolarize in response to NMDA, the extent of Ca^++^ influx through NMDA channels and various other routes will vary in a complex manner [66]. However, some of the initial stiffening might be attributed to increased contractile tone due to Ca^++^-mediated actomyosin interactions (see 39,59,67).

The uncompromised plasma membrane is a far more complex structure than a pathological bleb, an entity lacking a contractile cortex. All else being equal, a swelling cell will stiffen as it becomes more turgid/rounded. If, in the neurons studied here, cortical actomyosin activity increases with osmotic swelling (evident in molluscan neurons [60]), this would augment hydrostatic pressure (which is also the physiological force for locomotory bleb extension [28]) and thereby augment measured elasticity. However, within those first minutes, increasing [Ca^++^]_int_ should begin to overactivate Ca^++^-proteases. The resulting degradation of the membrane skeleton could account for the secondary softening we observed. NMDA-mediated Ca^++^-influx is known to activate gelsolin within minutes [68] while the impacts of neuronal calpain activation develop more slowly [69]. In the long Nav-rich axon initial segments of cortical neurons, leaky/overactive Nav channels (NMDA channels blocked by MK801) trigger calpain-dependent destruction within 2 hours of cerebral oxygen-glucose deprivation [70].

Though neurons are among the softest of cell types (e.g. >10-fold softer than human keratinocytes [71,72]), characteristic elasticity differences [43] are discernible among different neuronal subtypes. For example, the elasticity of dorsal root ganglion neurons is 3.2 times higher than that of rat cortical neurons (in regions of the cortex with medium elasticity) as determined by a force mapping study [73]; the average elasticity there, 163 Pa, is similar to that measured here for cultured cortical neurons. It is notable that when the most rigid and most soft cell types (keratinocytes and neurons) undergo cell-compression induced blebbing, the blebbed-out membranes have the same small Young’s moduli [72]. For intact plasma membranes, it is harder to characterize a “pure” elasticity, as shown by [74]: when deforming intact cultured cortical neurons by AFM, indentations report on cortex-mechanics with contributions from nucleus-mechanics. For this reason, we emphasize within-preparation elasticity differences (ratios) more than absolute values. Moreover, the neuron plasma membrane has reversible access to tension-sensitive bilayer reserves [59,60,67,75], and so might be expected to exhibit loading-rate dependent Young’s moduli. Whether this contributes to the loading-rate dependent Young’s moduli reported for cortical neuron by Bernick et al. [74] is unknown.

Particularly germane to the present work are several studies on non-neuronal cells that queried how ionic and osmotic fluxes modulate cell elasticity. Steltenkamp et al. [76] exposed confluent MDCK-II cells to hypo- and hyper-osmotic media, separately monitoring cell volume by Z-plane confocal microscopy and elasticity measurements by AFM force spectroscopy. Cells made to shrink or swell (15 min exposure to 920/330 or 170/330 mosM-media, respectively) were stiffer or softer respectively than cells in control (330/330) medium. The interpretation, namely that cortical F-actin is the major determinant of elasticity, and that detachment of the F-actin cortex from the plasma membrane (i.e., bleb formation) causes softening (depicted in their Scheme 1), accords with other AFM-based findings involving actin reagents [42,72,77]. Sachs and colleagues [78], who exposed astrocytes and diverse cell lines to more severely hyposmotic media (90/340) than Steltencamp et al [76], also observed softening, presumably due to severe blebbing. While assorted theories have been proposed to explain the softening that attends bleb-damage [42,76,78], a simple interpretation may suffice if we assume that mechanically, a bleb is like a liposome large enough to render bilayer bending elasticity inconsequential (see 79); it will be softer when flaccid (non-spherical), stiffer (turgid) when approaching sphericity. For undifferentiated mammalian cells Spagnoli et al. [78] have confirmed experimentally that inflating a grossly blebbed cell by fluid injection stiffens the cell; the Young’s modulus simultaneously determined by AFM increased sharply.

Sanchez et al. [80] probed DRG neuronal mechanics via scanning ion conductance microscopy. We infer, based on their interpretive Figure 8, (part F, showing almost no adhesive contacts between cortical proteins and bilayer), that their view is that the neuronal somata became blebbed under the influence of negative pipette pressure (aspiration) (see also 24), and that the bleb accounted for the soft zone above the more rigid core of the neuron. Pipette aspiration causes traumatic bleb-type damage in molluscan neurons, such that successive bouts of aspiration render it easier for mechanosensitive K^+^ channels to respond to bilayer stretch [64]. In those neurons, osmotic swelling, Ca^++^ ionophore, and cytochalasin all produce this same effect [17].

In summary, the findings presented here indicate that dissection/identification of molecular events during the excitotoxic transition from stiff/swollen to soft/blebbing is warranted and should be feasible. Future studies could exploit the biphasic AFM response to provide information on the steps that occur during the transition from mild reversible to severe irreversible excitotoxic damage. In brain slices it may be possible to probe differences in the cytomechanical responses to agonist exposure in injury-vulnerable and injury-resistant [81] CA1 versus CA3 hippocampal neurons or differences in the cytomechanical responses to ischemic anoxic depolarization in ischemia-vulnerable cortical neurons versus ischemia-resistant hypothalamic neurons [82].

## Supporting Information

Figure S1 **Fluorescence and AFM images of immunostained and fixed neuron cultures.** (A) GFAP (green) and MAP-2 (red) immunostaining are used to visualize both neurons and astrocytes. (B) MAP-2 staining only. (C, D) Correlated fluorescence (MAP-2 staining) and AFM images of one neuron; AFM was measured with a small tip, not the large spherical tip used for force indentation curves.
(TIF)

Figure S2 **Additional elasticity measurements.** (A) Approach and retraction curves measured on neuron soma. (B) Histograms of Young’s modulus obtained for 4 individual neuron somata from a single cell culture. Each neuron is displayed in a different color.
(TIF)

Figure S3 **Mechanical response of neurons to 20 µM NMDA.** Young’s modulus measurements of 3 individual neurons before (green, blue) and after (red, measured ~50 min after addition) treatment with NMDA.
(TIF)

Figure S4 **Brightfield optical images of neurons before and after treatment with** 20 µM **NMDA**. (A) Brightfield optical image of a neuronal culture before NMDA treatment. (B, C) Images of the neuron highlighted in image A, before (B) and after (C, 4 hr) NMDA treatment. (D, E) Images from a different culture plating before (D) and after (E, 1 hr) NMDA treatment. The “after” images illustrate the increased size of the neurons after exposure to NMDA. Images A, D and E are 224 x 168 µm^2^.
(TIF)

Figure S5 **Control experiment showing that BPS exchange did not affect neuronal elasticity.**
(TIF)

Figure S6 **Mechanical response of neuron somata upon exposure to 12.5 µM NMDA.** Each color of symbol represents an individual neuron soma and the data are displayed as E/E_0_. Washout times are indicated with color-coded arrows on the x-axis. The dashed line indicates the initial pretreatment value.
(TIF)
