# Supplementary figures and images for: Force Spectroscopy Measurements Show That Cortical Neurons Exposed to Excitotoxic Agonists Stiffen before Showing Evidence of Bleb Damage
Source: PLoS One. 2013 Aug 30;8(8):e73499. doi: 10.1371/journal.pone.0073499 (PMC3758302; doi:10.1371/journal.pone.0073499)

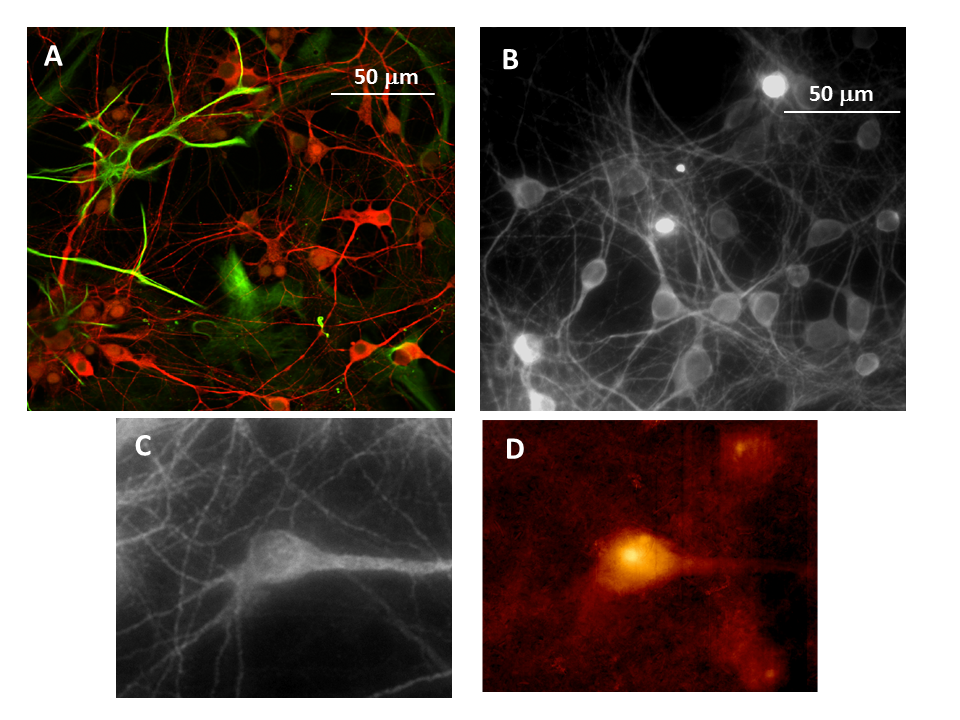

Supplement: Figure S1 — Fluorescence and AFM images of immunostained and fixed neuron cultures. (A) GFAP (green) and MAP-2 (red) immunostaining are used to visualize both neurons and astrocytes. (B) MAP-2 staining only. (C, D) Correlated fluorescence (MAP-2 staining) and AFM images of one neuron; AFM was measured with a small tip, not the large spherical tip used for force indentation curves. (TIF) [file pone.0073499.s001.tif]

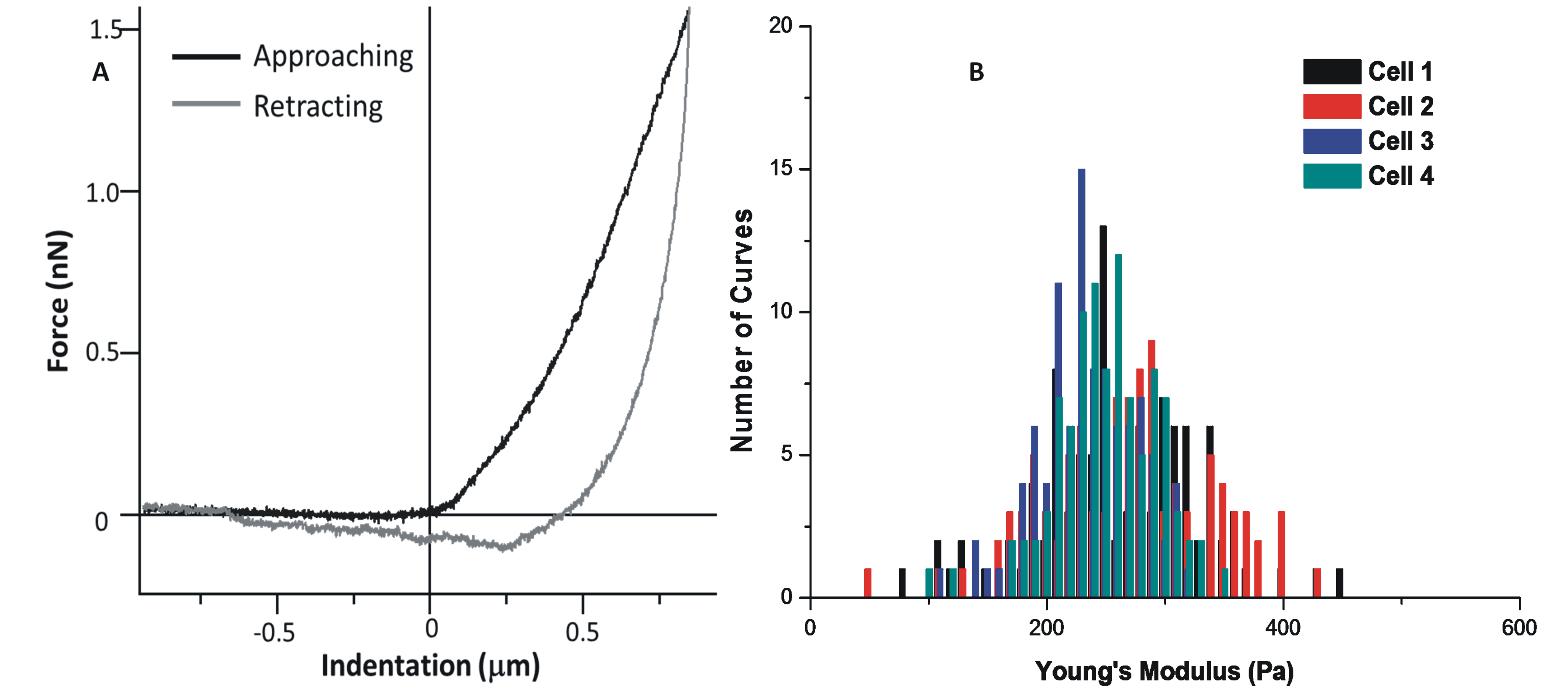

Supplement: Figure S2 — Additional elasticity measurements. (A) Approach and retraction curves measured on neuron soma. (B) Histograms of Young’s modulus obtained for 4 individual neuron somata from a single cell culture. Each neuron is displayed in a different color. (TIF) [file pone.0073499.s002.tif]

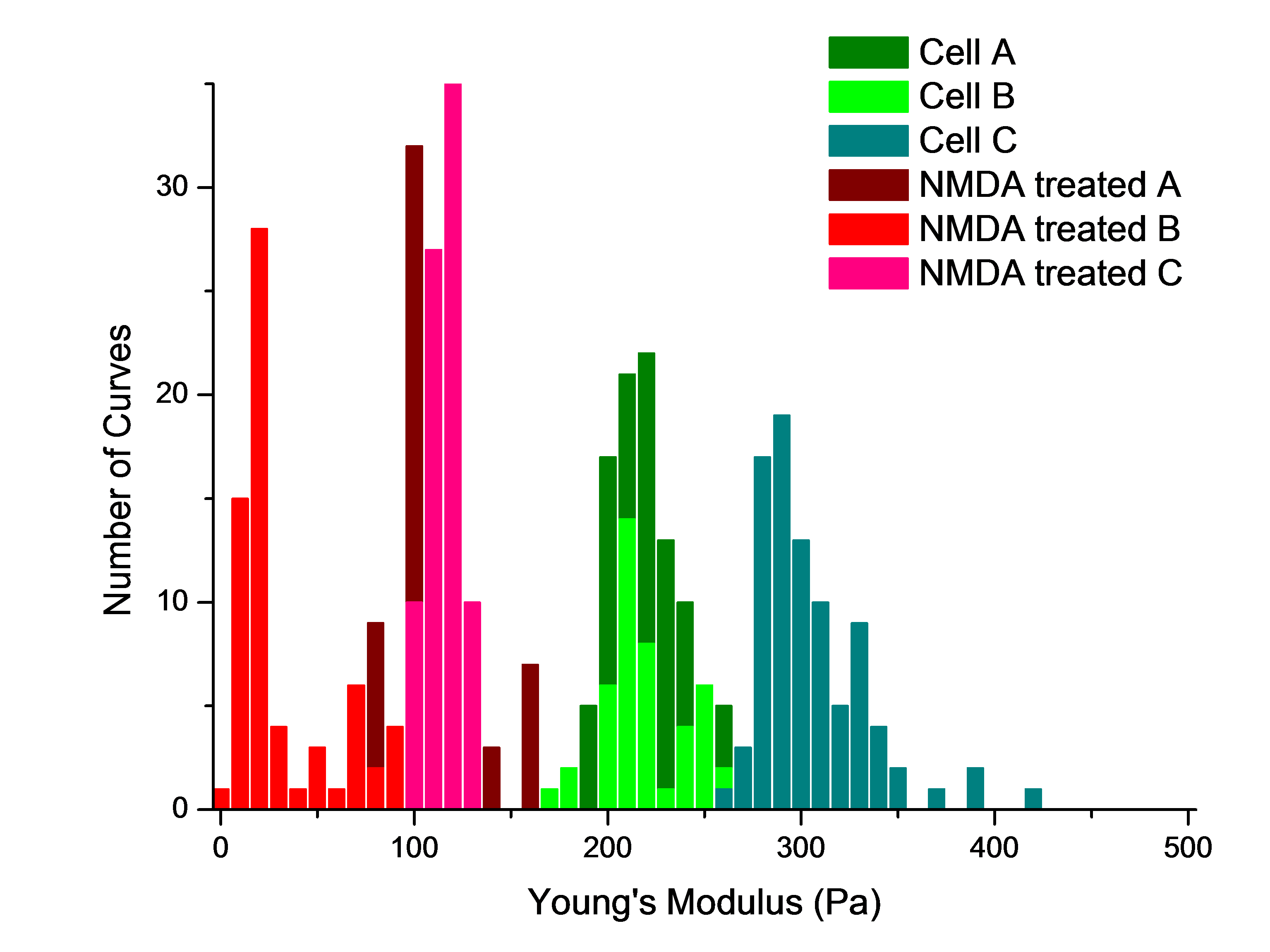

Supplement: Figure S3 — Mechanical response of neurons to 20 µM NMDA. Young’s modulus measurements of 3 individual neurons before (green, blue) and after (red, measured ~50 min after addition) treatment with NMDA. (TIF) [file pone.0073499.s003.tif]

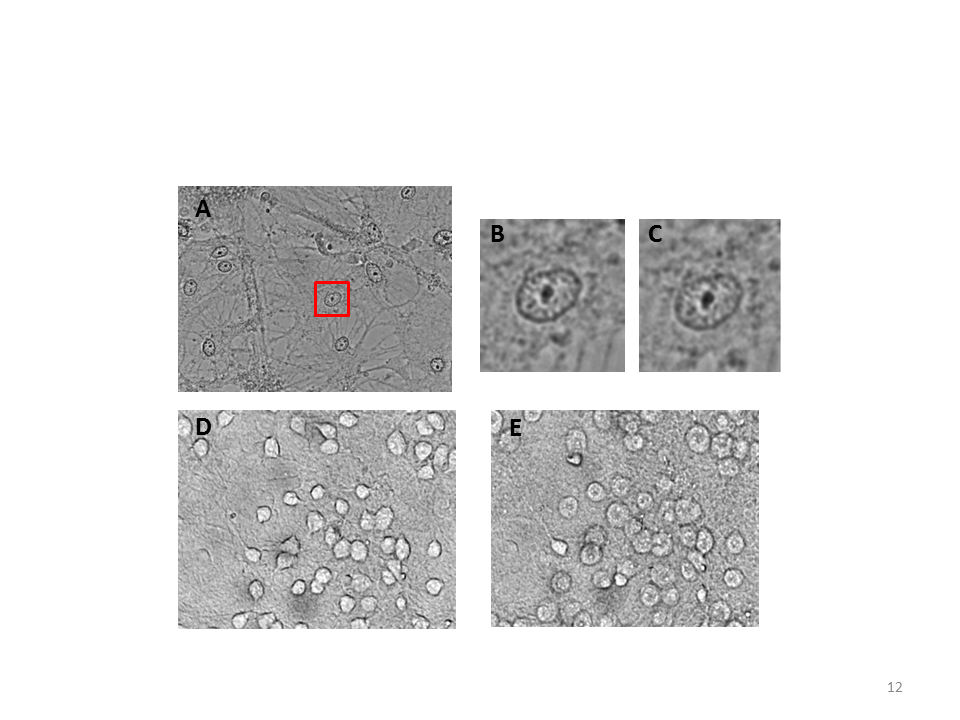

Supplement: Figure S4 — Brightfield optical images of neurons before and after treatment with 20 µM NMDA. (A) Brightfield optical image of a neuronal culture before NMDA treatment. (B, C) Images of the neuron highlighted in image A, before (B) and after (C, 4 hr) NMDA treatment. (D, E) Images from a different culture plating before (D) and after (E, 1 hr) NMDA treatment. The “after” images illustrate the increased size of the neurons after exposure to NMDA. Images A, D and E are 224 x 168 µm2. (TIF) [file pone.0073499.s004.tif]

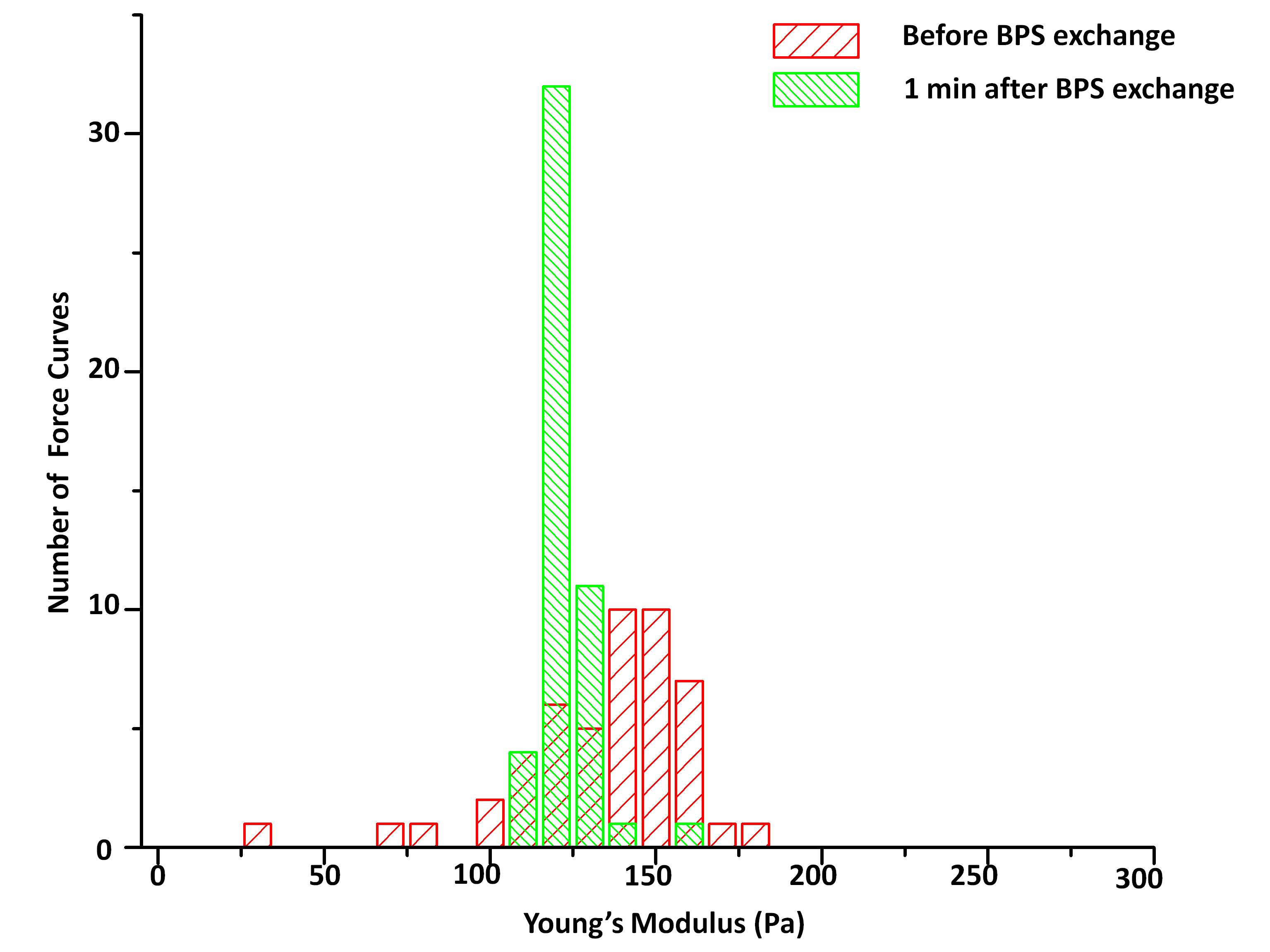

Supplement: Figure S5 — Control experiment showing that BPS exchange did not affect neuronal elasticity. (TIF) [file pone.0073499.s005.tif]

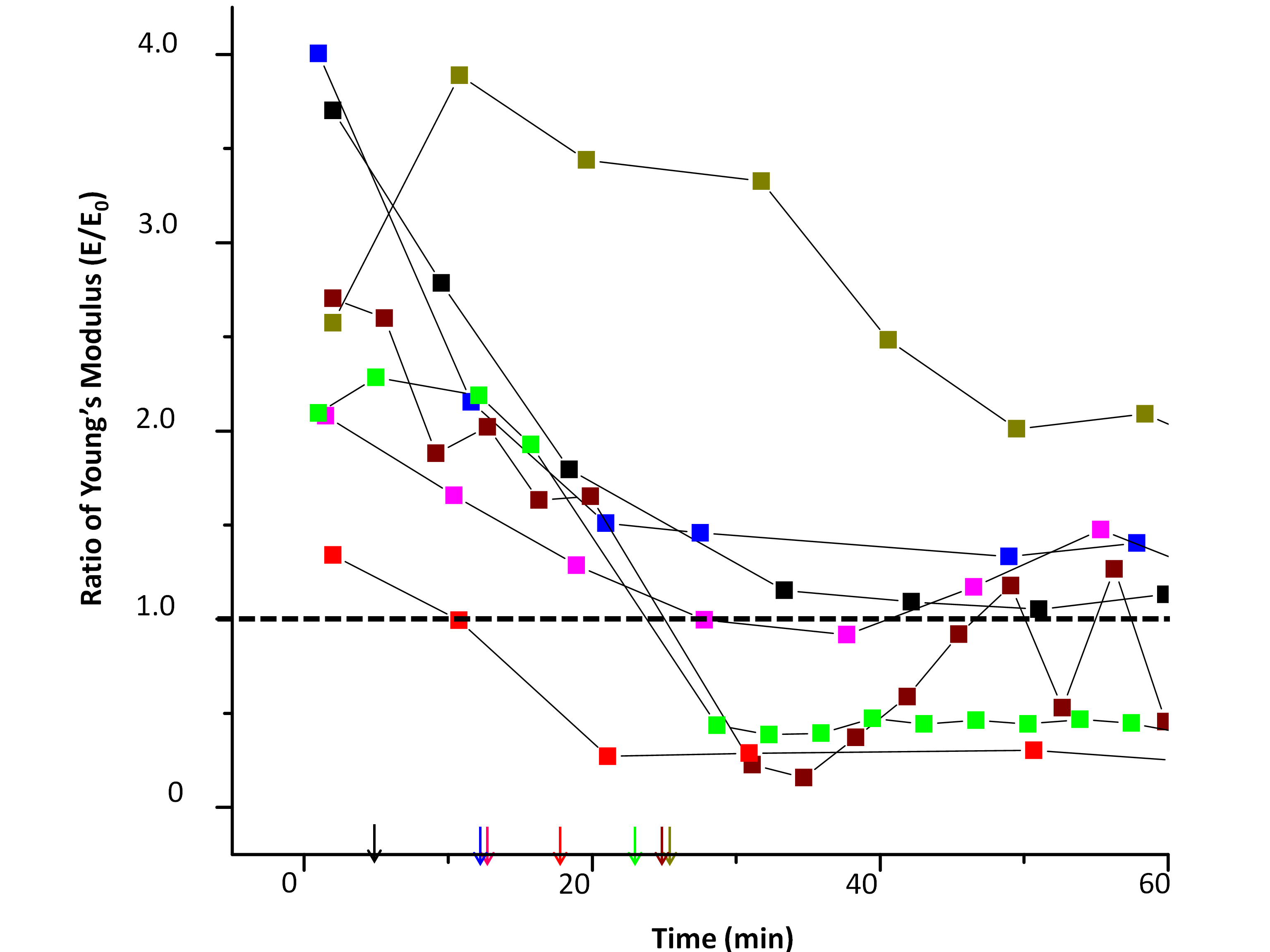

Supplement: Figure S6 — Mechanical response of neuron somata upon exposure to 12.5 µM NMDA. Each color of symbol represents an individual neuron soma and the data are displayed as E/E0. Washout times are indicated with color-coded arrows on the x-axis. The dashed line indicates the initial pretreatment value. (TIF) [file pone.0073499.s006.tif]
